# Co-expression Network Analysis Elucidated a Core Module in Association With Prognosis of Non-functioning Non-invasive Human Pituitary Adenoma

**DOI:** 10.3389/fendo.2019.00361

**Published:** 2019-06-06

**Authors:** Busra Aydin, Kazim Yalcin Arga

**Affiliations:** Department of Bioengineering, Marmara University, Istanbul, Turkey

**Keywords:** co-expression, differential co-expression network, non-functional pituitary adenoma, invasiveness, prognosis, biomarker

## Abstract

Non-functioning pituitary adenomas (NFPAs) are tumors with clinically challenging features since they have insidious progression. A complex network of gene interactions is thought to have roles in tumor formation and progression. Therefore, revealing the genetic network behind NFPA tumorigenesis is not only essential to attain further knowledge of tumor biology, but also plays a fundamental role in the development of efficacious treatment strategies. Differential co-expression network analysis is an outstanding approach for elucidation of groups of genes which show distinct co-expression patterns among phenotypes. In this study, we carried out a differential co-expression network analysis of NFPA-associated transcriptome dataset (*n* = *40*) considering invasive (*n* = *22*) and non-invasive (*n* = *18*) phenotypes. Furthermore, we identified differentially co-expressed and co-regulated mRNA modules, which might be considered as potential systems biomarkers for NFPA prognosis and invasiveness. As a result, we have identified a novel 13-gene module, including *CEACAM6, CYP4B1, EIF2S2, HID1, IFFO1, MYO18A, PDCD2, SGIP1, SWSAP1*, and four unknown genes (*A_24_P127621, A_24_P255786, A_24_P683553*, and *A_24_P916979*), which was able to categorize the patients into two groups as invasive and non-invasive NFPA with distinct prognosis. The prognostic core module genes were associated with progression and prognosis of brain and glandular based cancers as well. Furthermore, these module genes were also expressed in blood, salivary gland, and spinal cord tissues. These results may provide the evidence on featured gene module which might play a prominent role in NFPA prognosis and sub-typing as effective biomarkers and therapeutic targets in the future.

## Introduction

Pituitary adenomas (PAs) are the second most frequently reported primary brain tumors that are associated with increased mortality and morbidity (1, 2). Non-functioning pituitary adenomas (NFPAs) are the most common type of PAs that are not hormonally active (3). In contrast with the functioning pituitary adenomas (FPAs), which secrete excess levels of hormones that can lead easily to track endocrine syndromes, the detection of pituitary adenomas including NFPAs is clinically challenging. They are diagnosed usually in the context of mass effect (compress adjacent neurovascular tissues) leading to visual loss, dysfunction of the pituitary, and cranial neuropathies. PAs are usually non-invasive and benign neoplasms in nature, but some of them exhibit an aggressive attitude and represent invasive characteristics through cells from a neoplasm extend to the adjacent healthy tissues and infiltrating into them. Invasive PAs display local invasion, elevated risk of postoperative recurrence, and inadequacy of therapeutic response (4). Even in the perturbation of surrounding tissues, invasive PAs are not considered as malignant.

In the classification of tumors of the endocrine system, World Health Organization (WHO) offers several markers associated with invasiveness such as the Ki-67 proliferative index, the number of mitotic figures, and the expression of p53 (5). Moreover, FSH-β, LH-β, and/or α-subunit immunostain positivity and expressivity of SF-1, GATA-2, and ER-α transcription factors were proposed as the indicators of the gonadotroph lineage in NFPAs, and null cell adenomas were defined as negative immunoreactivity for both pituitary hormones and pituitary transcription factors (5, 6).

The expression profiling studies at RNA level intending the identification of molecular markers associated with NFPA invasiveness are very limited. The upregulation of *MYO5A*, and downregulation of E-cadherin (*CDH1*) and H-cadherin (*CDH13*) were proposed as markers of invasiveness at mRNA level (7, 8). Several microRNAs including miR-135a, miR-140-5p, miR-582-3p, miR-582-5p, and miR-938 were found as overexpressed in the invasive state, and they were also associated with tumor size and tumorigenesis (9). Moreover, the expression levels of lncRNAs, maternally-expressed gene 3 (*MEG3*) and Hox transcript antisense intergenic RNA (*HOTAIR*), were correlated with NFPA development and invasion (10).

Despite the proposed molecular markers and immunohistochemical indicators of invasiveness, early prediction of invasive NFPA remains considerably arduous since there is no consistent pattern that differentiates invasive from non-invasive PA. Thus, invasive PA needs to be properly defined with molecular markers in order to identify patients at increased risk of early recurrence or subsequent tumor progression. The present markers and classification systems are insufficient to guide precise diagnostic and therapeutic decisions in pituitary tumor invasiveness.

Differential co-expression is defined as the altered co-expression patterns of genes between two phenotypes and represents significant potential to identify gene clusters associated with the phenotype of interest (11). In several studies, co-expression analyses were performed to elucidate the PA metastasis, invasion and progression. Zhang et al. identified co-expressed gene pairs for prolactin (PRL) secreting pituitary tumor metastasis and hypothesized that *SLC2A11, TENM1, IPO7*, and *CHGB* are associated with metastasis in prolactinoma cases (12). Co-expression of somatostatin and dopamine receptors were also investigated and invariable loss in expression of both receptors in invasive growing corticotroph adenomas was reported (13).

In this study, we aimed to determine whether invasive and non-invasive PAs display differences in mRNA expression profiles through a differential co-expression network analysis framework. A novel gene module, which was differentially co-expressed in non-invasive PA when compared to invasive PA state, was presented, and its prognostic performance was tested in several tumor types through survival analyses. Topological and functional enrichment analyses were performed to elucidate the molecular mechanisms. Transcriptional regulatory elements (i.e., transcription factors and miRNAs) associated with the prognostic gene module were investigated, and expression levels of the module genes in various tissues were also screened.

## Methods

### Selecting Gene Expression Profiling Dataset

In order to gather NFPA associated gene expression profiling datasets, we comprehensively screened the publicly available functional genomics data repositories including ArrayExpress (14), NCBI Gene Expression Omnibus (GEO) (15) and The Cancer Genome Atlas (TCGA, http://cancergenome.nih.gov/). Among three transcriptome datasets, GSE63357 (16), GSE77517 (17), and E-TABM-899 (7), carried out with NFPA samples (with no hormone secretion), we preferred to employ E-TABM-899 considering its large sample size and sampling characteristics. Expression profiles of mRNA and related clinical data of invasive and non-invasive PA were downloaded from ArrayExpress database (https://www.ebi.ac.uk/arrayexpress/). Gene expression profiles generated using Agilent whole human genome oligo microarrays were used as a training set to construct co-expression networks, identify hub genes, and differentially expressed genes (DEGs) in this study. Transcriptome data was composed of 40 samples classified as invasive (*n* = 22) or non-invasive (*n* = 18). NFPA samples were characterized in terms of clinical and pathological features including age, sex, hormonal secretion (LH, FSH), immunohistochemical staining, tumor volume (varied between 0.5 and 77 cm^3^), tumor grade, follow-up time, and outcome (recurrence, stably remnant, and remnant). Immunostaining for FSH-β and LH-β and/or α-subunit was used for tumor characterization. According to WHO 2017 classification of tumors of the pituitary gland (5), the dataset was composed of 38 samples with gonadotroph lineage (positive immunostains FSH-β and LH-β and/or α-subunit) and 2 samples with null cell adenoma (negative for immunostains) (5, 6). Tumor size and invasiveness were determined with regard to preoperative magnetic resonance imaging and perioperative findings, by employing the modified Hardy criteria (18), which is grade I, if tumor < 10 mm, it is enclosed microadenoma; grade II, if tumor > 10 mm, it is enclosed macroadenoma; grade III, localized perforation of the sellar floor; and grade IV, diffusive destruction of the sellar floor. Tumor recurrence was classified according to radiological findings of re-growth of the tumor remnant or new tumor growth (in patients with no surgical remnants). Remnant tumors were accepted as stable if there was no sign of growth on two MRI within the 1-year interval and no clue of disease reactivation. Follow-up times were years that passed after surgery and varied between 3 and 10.5 years. This dataset included tumors in varied grades, 1 of grade I, 18 of grade II, 6 of grade III, and 15 of grade IV. Grade III and IV tumors, as well as one Grade II tumor exhibiting extradural extension, were accepted as invasive.

### Data Processing and Identifying Differentially Expressed Genes

Pre-processing of microarray data was performed in R using expression profiles (E-TABM-899.eSet.r) downloaded from ArrayExpress database (14). The expression profiles were normalized through quantile normalization. DEGs between invasive and non-invasive PAs were identified from the normalized expression values by using Linear Models for Microarray Data (LIMMA) package (version 3.34.5) (19). The Benjamini-Hochberg method was used to control the false discovery rate. In addition, cross-validation of the DEGs was performed using the two-sample *t*-test. To determine the statistical significance, adjusted *p* < 0.05 was used, and genes that showed at least 10% change in expression were selected for further consideration in the network construction.

### Pathway and Functional Enrichment Analysis for DEGs

The pathway and functional enrichment analyses were performed using ConsensusPathDB (Release 32) (20) to identify functional annotations significantly associated with the DEGs. The Kyoto Encyclopedia of Genes and Genomes (KEGG) (21) database was preferably used as the pathway database. The Gene Ontology (GO) terminology (22) was employed as the source for annotating the molecular functions and biological processes. *P*-values were obtained via Fisher's Exact Test. Benjamini-Hochberg's correction was used as the multiple testing correction techniques, and enrichment results with adjusted *p* < 0.01 were considered statistically significant. Comparison of the significant GO terms and KEGG pathways was performed to identify the different biological functions and processes between the invasive and non-invasive samples.

### Co-expression Network Construction for Invasive vs. the Non-invasive States

Co-expression networks were constructed for invasive and non-invasive PA states as previously described (11). Initially, among the 29,754 genes detected in the pituitary samples, DEGs were identified. In the case of the repetition in expression values of DEGs, the mean expression values were computed and used in further analyses. Pearson's correlation coefficient (PCC) was employed to determine the co-expression levels between DEG pairs in each state. Benjamini-Hochberg's correction was used as the multiple testing correction techniques, and the gene pairs were regarded as co-expressed if |PCC| ≥ 0.80 and adjusted *p* ≤ 0.05.

To identify differential co-expression profiles between two states, the following criterion was employed:

$$\begin{array}{l} |\left({\text{PCC}}_{\text{NONINV}}-{\text{PCC}}_{\text{INV}}\right)/{\text{PCC}}_{\text{INV}}|\geq 1 \end{array}$$

where PCC_NONINV_ and PCC_INV_ are the PCC of a DEG pair in non-invasive and invasive states, respectively. Resultant gene pairs were selected to construct the co-expression networks, ICON (invasive co-expression network) and NICON (non-invasive co-expression network), representing invasive and non-invasive states of PA, respectively.

### Identification of Differentially Co-expressed Gene Modules

To elucidate highly connected network modules, co-expression networks were analyzed using MCODE (23) plugin of Cytoscape (v.3.5.1) (24). Modules with at least 10 nodes (genes), average connectivity ≥10 and clustering coefficient ≥ 0.5 were considered as differentially co-expressed modules between invasive and non-invasive states. Modules were further analyzed with Network Analyzer (25) and Cytohubba (26) plug-ins to determine the significantly altered hub genes with respect to local and global topological metrics (i.e., degree and betweenness centrality).

### Sub-type Clustering Performance of Co-expressed Gene Modules

Considering the expression profiles of module genes, Principal component analysis (PCA) was performed to cluster invasive and non-invasive samples. The principal components explaining at least 85% of the total variance was considered in the determination of clustering performance, i.e., the ability hereupon “sub-typing” of PA.

### Prognostic Power and Survival Analyses

PCA was performed based on gene expression profiles of module genes, and samples were categorized into three clusters using k-means algorithm taking into consideration the first three principal components (explaining 98% of the total variance). Cox regression was performed via MedCalc Statistical Software (v.18.2.1, Ostend, Belgium) to elucidate survival-associated genes. Analysis results were visualized and compared through Kaplan-Meier plots using the follow-up times of invasive and non-invasive groups. The prognostic power of the module was determined using the log-rank test *p*-value, the hazard ratio (HR), and its confidence intervals (CI).

### *In silico* Validation in Related Tumor Types

We used SurvExpress (27) web-based biomarker validation tool in order to test the validity of the proposed prognostic gene module in various cancer types. Validation analyses were carried out through independent RNA-seq datasets obtained from TCGA (28) and microarray datasets from NCBI-GEO. Core module genes were tested through glandular based cancers including adrenocortical carcinoma (TCGA, *n* = 77), breast invasive carcinoma (TCGA, *n* = 502), and ovarian serous cystadenocarcinoma(TCGA, *n* = 247); brain tumors including glioblastoma (TCGA, *n* = 660), meningioma **(**NCBI-GEO, GSE16581, *n* = 68), and medulloblastoma (NCBI-GEO, GSE37418, *n* = 76) (29, 30); and other cancers including prostate adenocarcinoma (TCGA, *n* = 497) and lung adenocarcinoma (TCGA, *n* = 475). For each dataset employed, samples were partitioned into low and high-risk groups according to their prognostic index, survival multivariate analyses, and risk assessments. The prognostic performance of modules in each dataset was determined using Kaplan-Meier plots, log-rank test *p*-values, hazard ratios (HR), and their confidence intervals (CI). Differences in gene expression levels between high and low-risk groups were presented by box-plots and statistical significance of the difference was calculated through *t*-test. Heatmaps were also created to clarify the correlation of the survival analysis with gene expression levels. Samples were sorted by their prognostic index and genes were clustered by using Euclidean distance.

### Screening of Gene Expressions in Varied Tissues

The expression levels of modules genes in easily collectible tissues were obtained from The Genotype-Tissue Expression (GTEx) project (31), which provides a genome-scale expression profiling (RPKM) of 51 normal human tissues, cells, and fluids.

## Results

### Transcriptional Profiling in Non-functioning Non-invasive Human Pituitary Adenoma

Transcriptome data (E-TABM-899) was composed of 40 NFPA samples, which were characterized in terms of clinical and pathological features including age, sex, hormonal secretion (LH, FSH), immunohistochemical staining (FSH-β and LH-β and/or α-subunit), tumor volume (0.5–77 cm^3^), tumor grade (I-IV), follow-up time (3–10.5 years), and outcome (recurrence, stably remnant, and remnant). Tumor size and invasiveness were determined with regard to preoperative magnetic resonance imaging and perioperative findings, by employing the modified Hardy criteria (18), Tumor recurrence was characterized according to radiological findings of re-growth of the tumor remnant or new tumor growth (in patients with no surgical remnants). As a result, the samples were classified into invasive (*n* = 22) and non-invasive (*n* = 18) groups.

**Figure 1:**
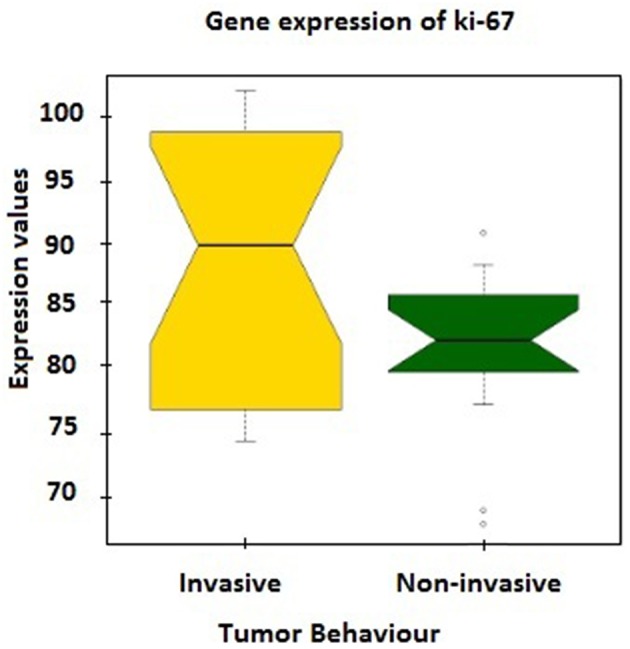
The expression values of MKI67 gene, which encodes Ki-67 protein, between tumor groups.

Further verification of sample classification was carried out employing the expression values of MKI67 gene, which encodes Ki-67 protein accepted as a labeling index of proliferation (32). MKI67 expression levels in two groups were compared and a statistically significant difference (*p* = 0.02) was observed between mean expression levels of invasive (88.49 ± 2.32) and non-invasive groups (81.62 ± 1.56; Figure 1).

### Identification and Functional Annotation of Differentially Expressed Genes (DEGs)

Following up a standard protocol including quantile normalization of raw data, statistical comparisons of expression levels through “limma” package, and multiple testing correction with Benjamini-Hochberg method, a total of 4,091 genes were identified as differentially expressed between two phenotypes, i.e., invasive and non-invasive pituitary adenoma, considering a statistical confidence level of *p* < 0.05. In addition to *p*-values, we also considered at least 10% change in mean expression levels (FC < 0.9 for down-regulation, FC > 1.1 for up-regulation) as a criterion in the identification of DEGs and determination of their direction of regulation. This filtering resulted in 641 DEGs of which 145 were down-regulated and 496 were up-regulated.

**Figure 2:**
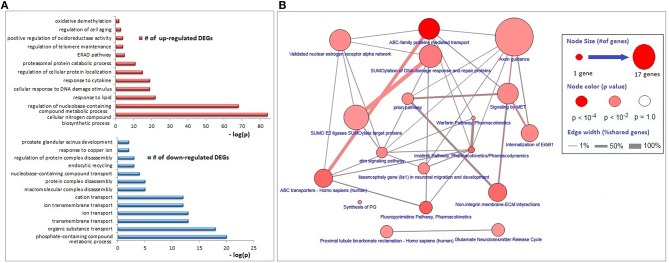
Biological process and pathway enrichments of DEGs. **(A)** Biological process enrichments of up- and down-regulated DEGs. **(B)** Pathway enrichments of DEGs.

The functional annotations of the down-regulated DEGs were significantly enriched with transmembrane transport of organic substances (especially the cations), disassembly of protein complexes and its regulation, endocytic recycling, and response to copper ion; whereas genes associated with responses to lipids, DNA damage stimulus, and cytokines, regulation of protein localization, proteasomal protein catabolism, endoplasmic-reticulum-associated protein degradation (ERAD) pathway, regulations of telomere maintenance and oxidoreductase activity, cell aging, and oxidative demethylation were up-regulated (Figure 2A). The major KEGG pathways significantly associated with DEGs (*p* < 0.01) were axon guidance (17 genes), ABC family mediated transport (8 genes), SUMOylation of DNA damage response and repair (6 genes), estrogen receptor alpha signaling pathway (5 genes), imatinib pathway (3 genes), and warfarin pathway (2 genes; Figure 2B).

### Detection of Differentially Co-expressed Gene Modules

**Figure 3:**
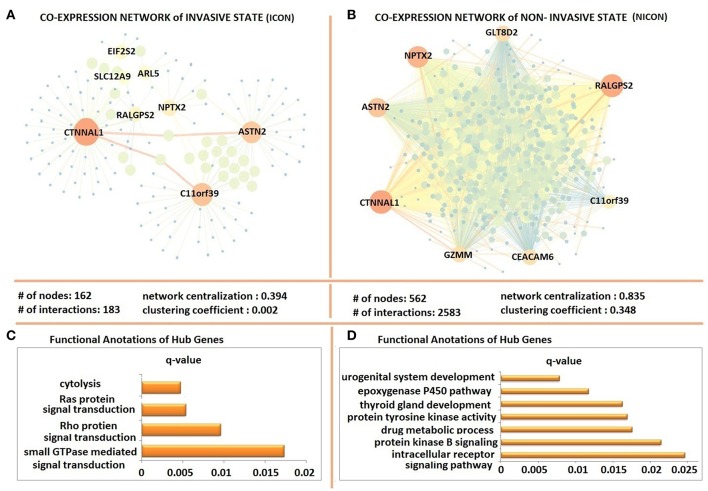
Differentially co-expressed gene networks of invasive and non-invasive states. **(A)** The co-expression network in invasive phenotype (ICON) with 183 links among 162 and topological features of ICON **(B)** the non-invasive co-expression network (NICON), which includes 2,385 links among 562 genes and topological features of the network **(C)** Functional annotations of ICON hub genes **(D)** Functional annotations of NICON hub genes.

The possible co-expression patterns of 641 DEGs were analyzed through the employment of PCCs in invasive and non-invasive phenotypes separately to construct phenotype-specific co-expression networks. This resulted with a denser, centralized, and modular co-expression network in invasive phenotype (ICON) with 183 links among 162 genes (Figure 3A) when compared to the non-invasive co-expression network (NICON), which includes 2,385 links among 562 genes (Figure 3B). Not only the number of genes exhibiting co-expression pattern was almost 4-fold higher in the non-invasive state, but also the network centralization (0.835) and the clustering coefficient (0.348) were higher in NICON when compared to those in ICON (0.394 and 0.002, respectively).

Hub genes of ICON network (*ARL5, ASTN2, C11orf39, CTNNAL1, EIF2S2, NPTX2, RALGPS2*, and *SLC12A9*) were enriched in cytolysis, Ras protein and Rho protein signal transduction and small GTPase signal transduction biological processes (Figure 3C). Hub genes of NICON network (*ASTN2, C11orf39, CEACAM6, CTNNAL1, GZMM, GLT8D2, NPTX2, RALGPS2*) were enriched in processes such as urogenital system development, epoxygenase P450 pathway, thyroid gland development, protein tyrosine kinase activity, drug metabolic processes, protein kinase B signaling, intracellular receptor signaling pathway (Figure 3D).

Comparative analysis of the phenotype-specific co-expression networks yielded with 10 differentially co-expressed gene modules (ranging in size from 5 to 69 genes), which were activated in non-invasive phenotype when compared to invasive. Differentially co-expressed gene modules were named as M1 (Module 1), M2, M3, M4, M5, M6, M7, M8, M9, and M10. Module sizes and their associations with biological processes and PA were tabulated (Table 1). M1 was composed of 41 genes and these genes were enriched in regulation of cell adhesion. M2 had 45 genes that were enriched in GTPase activator activity and nucleoside triphosphatase regulator activity. M3 included 14 genes that related to Actin filament binding and Ras protein signal transduction processes. M4 had 69 genes which were enriched in DNA damage checkpoint and regulation of cellular response to stress. There were 10 genes in M5 and enriched in DNA damage checkpoint process. M6 had 8 genes that were related with metal ion binding process. M7 included 8 genes that enriched in pyrophosphatase activity, hydrolase activity, and acting on acid anhydrides. M8, M9, and M10 included 5 genes that were enriched in nervous system development, Ras and GTPase mediated signal transduction, and protein complex binding, respectively. Core module included 13 genes which were enriched in ATPase activity and regulation of endocytosis. These modules have been associated with neither PA diagnosis nor PA prognosis in literature.

**Table 1:** Identified modules and their features.

**Module identifier**	**Number of module genes**	**Functional enrichments (*p*-value < 0.001)**	Relationship with PA^*^
M1	41	Regulation of cell-cell adhesion	Novel as a module
M2	45	GTPase activator activity, Nucleoside-triphosphatase regulator activity	Novel as a module
M3	14	Actin filament binding, Ras protein signal transduction	Novel for every module gene
M4	69	DNA damage checkpoint, Regulation of cellular response to stress	Novel as a module
M5	10	DNA damage checkpoint	Novel for every module gene
M6	8	Metal ion binding	Novel for every module gene
M7	8	Pyrophosphatase activity, Hydrolase activity, Acting on acid anhydrides	Novel for every module gene
M8	5	Nervous system development	Novel for every module gene
M9	5	Ras protein signal transduction, Small GTPase mediated signal transduction	Novel for every module gene
M10	5	Protein complex binding	Novel for every module gene

* *If stated as novel for every module gene, each module gene and module itself was reported for the first time in this study. If stated as novel as a module, some of the module genes were reported as a biomarker for PA but not reported as a whole module*.

To determine the possible associations of the identified co-expression modules with clinicopathological features (i.e., age, tumor size, tumor grade, hormonal status, follow-up time, and clinical outcome), we conducted the Principal Components Analyses (PCA) as well as Kaplan Meier survival analyses, and detected significant correlations with tumor grade and sub-type for several gene modules.

**Figure 4:**
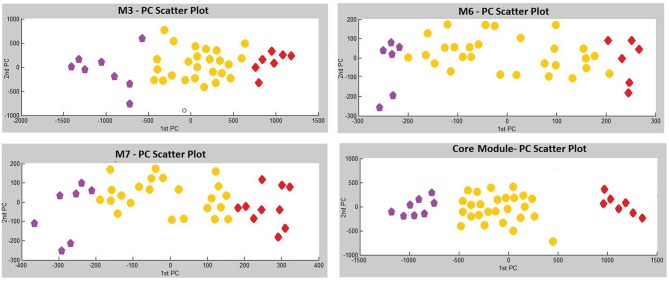
Principle Components Analysis (PCA) plots of differentially co-expressed gene modules (M3, M6, M7, and core module) that have significant prognostic performance.

Three modules, i.e., M3 with 14 genes associated with actin filament binding and Ras protein signal transduction, M6 with 8 genes associated with metal ion binding, and M7 with 8 genes associated with pyrophosphatase and hydrolase activities, presented considerable accuracy in discrimination of two sub-groups of invasive and non-invasive states in PA (Figure 4). Considering the similar discriminatory behavior of these modules, we combined them and narrowed the co-expression patterns with a higher PCC cut-off (increasing from 0.8 to 0.9) to obtain a “core module” consisting of the minimum number of genes with higher discrimination ability. This resulted with a core module of 13 genes (*CEACAM6, CYP4B1, EIF2S2, HID1, IFFO1, MYO18A, PDCD2, SGIP1, SWSAP1, A_24_P127621, A_24_P255786, A_24_P683553, A_24_P916979)*, which indicates higher discrimination accuracy, since clusters representing invasive and non-invasive states were clearly separable and distinguishable without any overlaps in PCA plots (Figure 4). Interestingly, the genes in the core module were not enriched in any molecular pathway or biological process as a whole; but some sub-groups were enriched in ATPase activity and regulation of endocytosis (*p* < 0.001). Notably, four genes (*A_24_P127621, A_24_P255786, A_24_P683553, A_24_P916979*) located in distinct chromosomes were not annotated with any molecular function yet (Table 2).

**Table 2:** Differentially co-expressed genes represented in the core module.

**Gene Name**	**Description**	**References**
CEACAM6	– A member of the immunoglobulin cell adhesion molecule superfamily. – Located on the cell surface of the mammary, respiratory and gastrointestinal epithelium and neutrophils. – Reported to be overexpressed in colorectal and pancreatic cancers.	(33–36)
CYP4B1	– An extrahepatic form of P450s. – Has a role in the bioactivation of xenobiotics – Strongly associated with bladder cancer.	(37, 38)
EIF2S2	– Has functions in encoding eukaryotic translation initiation factor -2 (subunit 2 beta) – Involved in early steps of protein synthesis.	(39)
HID1	– Associated with the medial- and trans- Golgi membranes. – Involved in the intracellular trafficking within the Golgi region.	(40)
IFFO1	– A member of the intermediate filament family. – Essential components of the cytoskeleton and nuclear envelope.	(41, 42)
MYO18A	– An unconventional member of the myosin superfamily. – Regulates epithelial cell adhesion and migration.	(43)
PDCD2	– Encodes a highly conserved nuclear protein, – Abnormal PDCD2 expression alters cell apoptosis. – Alteration of PDCD2 expression could be conducive to human cancer development and progression.	(44)
SGIP1	– One of the key regulators of clathrin-mediated endocytosis. – Functions as an endocytic protein that alters signaling by receptors in neuronal systems – Affects energy homeostasis via interaction with endophilins.	(45, 46)
SWSAP1	– Required for efficient homologous recombination repair in mammalian cells. – Has roles in the maintenance of genomic stability and tumor suppression.	(47)
A_24_P127621 A_24_P255786 A_24_P683553 A_24_P916979	– Genes with unknown functions.	

### The Prognostic Performance of the Core Module Between Different PA States

**Figure 5:**
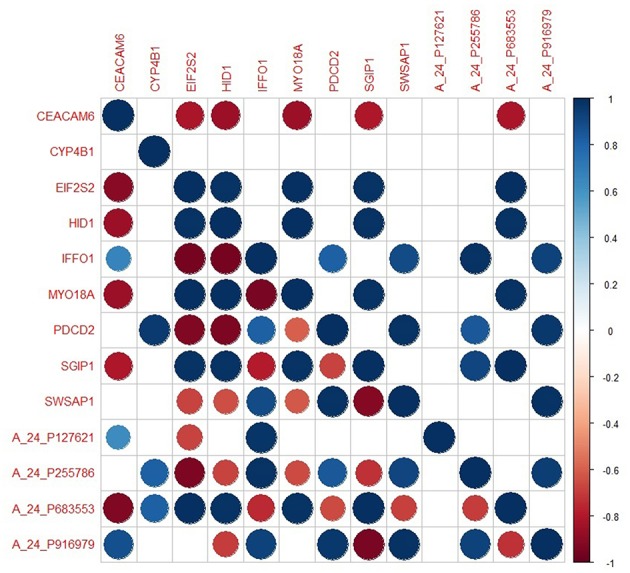
The degree of co-expression of core module genes between invasive and non-invasive states. Upper-triangle represents invasive state whereas lower-triangle shows non-invasive state.

**Figure 6:**
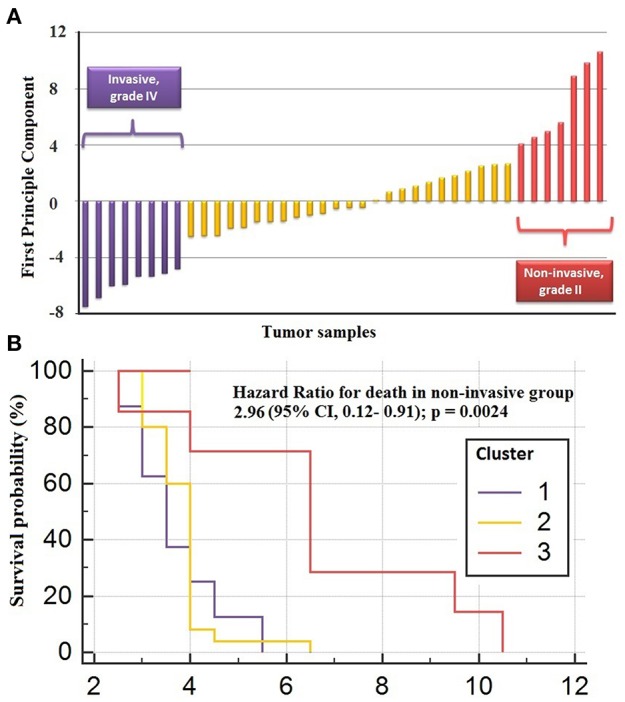
Clustering of sub-types of NFPA with PCA analysis. **(A)** The clustering of samples via first principle component of expression profiles of core module genes **(B)** survival analysis by Cox regression model and Kaplan-Meier estimates indicated the predictive power of patient survival among the sub-types.

The genes in the core module indicated significantly different co-expression patterns between invasive and non-invasive phenotypes of PA (Figure 5). The clustering of samples based on the expression profiles of core module genes via the first principle component (PC1) of the PCA analysis (describing 89.8% of the total variance) and k-means algorithm resulted with three distinct sub-groups of patients (Figure 6A). The cluster representing lower loadings on PC1 consisted of only invasive and grade IV tumor samples; whereas the cluster with higher loadings on PC1 included non-invasive and grade II tumors. Furthermore, survival analysis with the Cox regression model and Kaplan-Meier estimates indicated the predictive power of patient survival among the sub-types (Figure 6B). There was a significant difference among groups in terms of survival probability estimates (*p* = 0.0024, log-rank test), and the average survival probability of non-invasive and grade II tumors was significantly higher than that of invasive and grade IV tumors with a hazard ratio of 2.96 (*p* = 0.011).

### Transcriptional Regulators of the Core Module Genes

**Figure 7:**
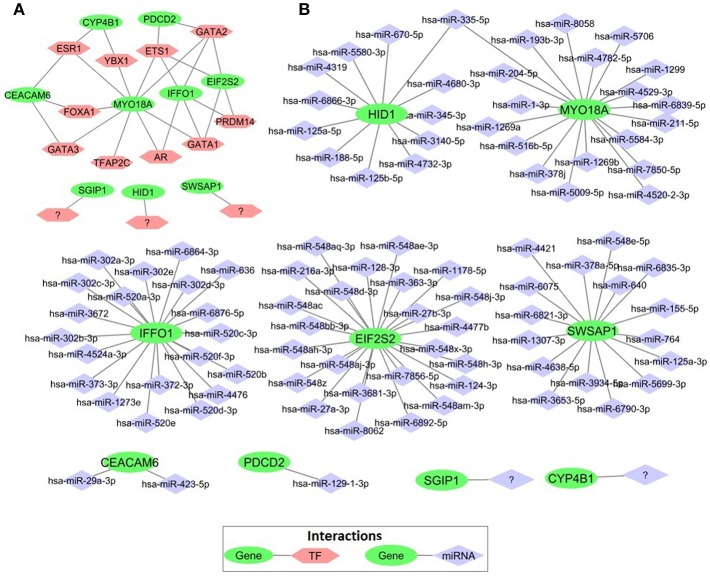
Transcriptional regulators of prognostic genes. **(A)** Transcription factors that regulate prognostic core module genes **(B)** microRNAs (miRNAs) regulating the core module genes.

In order to elucidate the regulatory mechanism behind the co-expression pattern of module genes and to evaluate the condition-specific expression pattern alterations, we performed serial analyses to link the key regulators of transcriptional control, i.e., transcription factors (TFs) and microRNAs (miRNAs), to genes in the core module. A total of 10 TFs were found as regulators of the module genes and *GATA1, GATA2, ETS1, ESR1*, and *PRDM14* were top five regulators with highest degree values (Figure 7A). The majority of the core module genes were co-regulated by *ETS1* and *GATA2*. Furthermore, among module genes *EIF2S2, IFFO1*, and *PDCD2* were co-regulated by the same TFs. In contrast to TFs, core module genes were regulated by distinct miRNAs (Figure 7B). Among those, mir-335-5p was the only common regulator between two gene pairs (*HID1* and *MYO18A*). Members of the core module were found in varied chromosomal locations. *CEACAM*6 and *SWSAP1* were located on chromosome 19 whereas *HID1* and *MYO18A* were on chromosome 17, *CYP4B1*, and *SGIP1* were on chromosome 1, *PDCD2* was on chromosome 6, A_24_P127621 was on chromosome 7, A_24_P683553 was on chromosome 9, A_24_P255786 was on chromosome 10, *IFFO1* was on chromosome 12, *EIF2S2* was on chromosome 20, and A_24_P916979 located on chromosome X.

### The Specificity of the Core Module to NFPA

**Figure 8:**
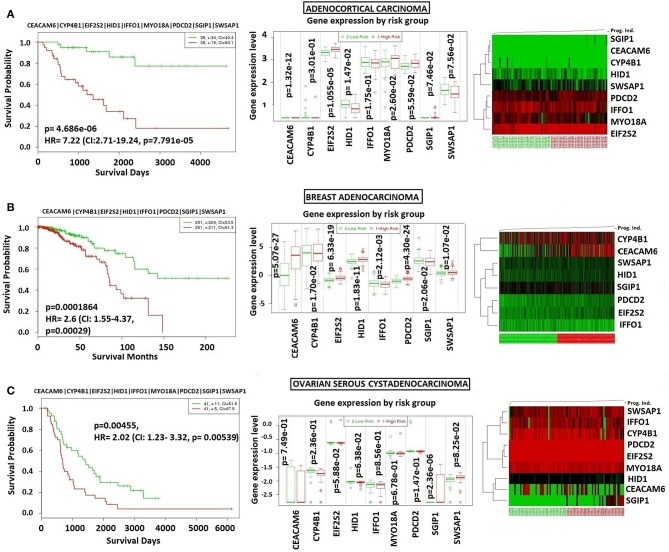
Prognostic power of core module through related tissue type cancers. Core module was also prognostic for **(A)** Adrenocortical carcinoma, **(B)** breast adenocarcinoma, **(C)** ovarian serous adenocarcinoma.

**Figure 9:**
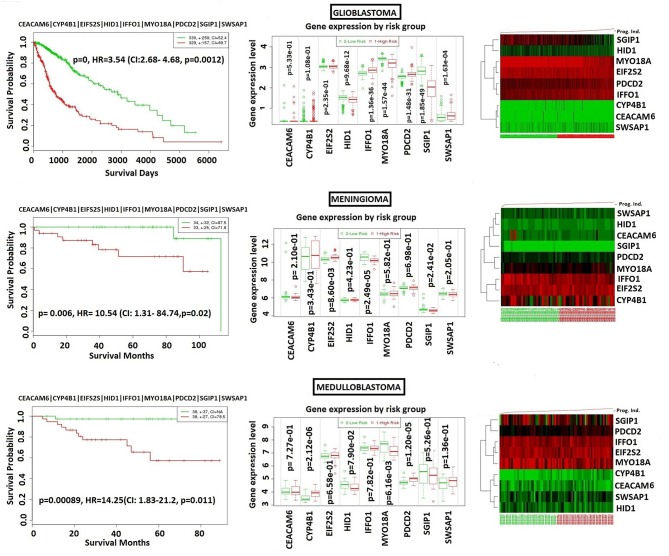
Prognostic power of core module through 3 types of brain tumors. **(A)** glioblastoma, **(B)** meningioma, **(C)** medulloblastoma.

In order to analyze NFPA-specific expression and prognostic performance of the core module, we carried out several analyses over various human cancers with or without tissue similarity with PA. First of all, we tested its prognostic performance in other glandular based cancers including adrenocortical carcinoma, breast invasive carcinoma and ovarian serous cystadenocarcinoma (Figure 8), and observed that the core module presents high prognostic performance in adrenocortical carcinoma (HR = 7.22, *p* = 4.69 × 10^−6^), breast invasive carcinoma (HR = 2.60, *p* = 1.84 × 10^−4^), and ovarian serous cystadenocarcinoma (HR = 2.02, *p* = 4.56 × 10^−3^). Secondly, we tested the performance of core module in brain tumors (Figure 9), and significant results were obtained in glioblastoma (HR = 3.54, *p* < 10^−20^), meningioma (HR = 10.54, *p* = 8.90 × 10^−4^), and medulloblastoma (HR = 14.25, *p* = 6.02 × 10^−3^). Lastly, we tested the prognostic performance of the core module in two other unrelated tissue but highly prevalent cancers, namely lung, and prostate adenocarcinoma. This time survival analyses pointed out insignificant performance in both lung (*p* = 0.08) and prostate (*p* = 0.15) adenocarcinomas (Figure S1). As a consequence, our core module could be considered as prognostic for both glandular-origin cancers and brain cancers, but not for lung and prostate cancers.

### Tissue-Specific Expressions of Core Module Genes

**Figure 10:**
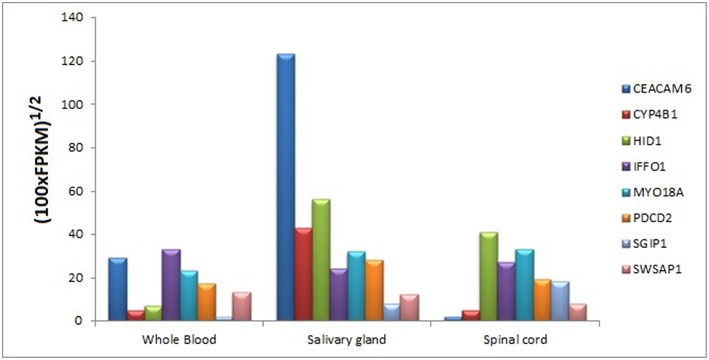
Tissue-specific expressions of core module genes.

We searched for the expression levels of module genes in easily collectible tissues (especially not need to biopsy or taking by minimally invasive operations) in order to decipher whether these genes can be detectable at mRNA level. Core module genes have detectable expression values in varied tissue types like blood, kidney, lung, spleen, spinal cord, saliva, pancreas, and uterus. It was also difficult to obtain a cumulative behavior in expression patterns of the genes in the same tissue; however, we found that every core module genes have considerable expression values in three tissue types, which are blood, salivary gland, and spinal cord (Figure 10).

## Discussion

There is a great need for interpretation of omics data to elucidate the multi-layered molecular mechanisms lay behind the invasion of PA. However, omics datasets associated with PA invasiveness is scarce in the publicly available databases. Even in TCGA, which is the most comprehensive and multi-dimensional database for cancer genomics with 33 cancer types, genomics and/or transcriptomics of PA were not considered. The scarcity of omics datasets indicates the urgent need for further efforts on mRNA profiling in NFPA.

Despite designated markers associated with invasive behavior of PA tumors such as the Ki-67 proliferative index, the number of mitotic figures, and the expression of *p53* (48, 49), the early prediction of PA invasiveness is still considerably hard, since the patient's state tends to be delayed due to the clinical symptoms of NFPA which are not obvious in the early stage. In the era of precision medicine, accurate biomarkers for cancer-specific prognosis and early prediction were urgently required and this convincingly enhances decision making for patient management.

Instead of the construction of the commonly used biological networks in literature like PPIs and metabolic networks, we constructed gene co-expression networks since it has several advantages such as almost complete coverage of human genes, little knowledge bias obtained from the published literature, and the ability to create cancer type–specific networks. Identification of changes in co-expression patterns of genes among invasive and non-invasive samples provides information about the invasiveness of PA-specific gene modules. Previous studies in literature objected to predict candidate prognostic biomarkers or protein-protein interaction networks constructed around differentially expressed genes in PA, not co-expressed gene modules. The altered co-expression schemes of module genes among invasive to the non-invasive state were neglected in these studies. Here, we made a differential co-expression analysis to elucidate PA genes and their co-expression pairs. As a result, the novel co-expressed gene module presented here may be regarded as “systems biomarkers” that lead to the design of effective therapeutic strategies in PA prognosis.

In the present study, co-expressed gene networks were constructed for invasive and non-invasive states (ICON and NICON, respectively) separately. ICON hub genes were enriched mostly in signaling processes like Ras, Rho, and small-GTPase signaling. It was reported that Ras small GTPase had a key signal transduction role in activating MAPK cascade (50). Rho GTPases are activated by G-protein-coupled receptors (GPCRs) and play critical roles in the invasion and metastasis of cancer cells (51). Hub genes of NICON were enriched in biological processes like urogenital system development, thyroid gland development, tyrosine kinase activity, protein kinase B signaling, and intracellular receptor signaling. The pituitary is part of the endocrine system and it was expected to found urogenital system development and thyroid gland development processes considering the secretion of TSH and FSH in the pituitary. Protein kinase B (Akt) signaling was found as over-expressed and over-phosphorylated in human PAs and proposed a potential role for Akt with respect to p27 deregulation (opposite direction to each other) (52).

The differential co-expression profiling followed by clustering and survival analyses resulted with a prognostic core module composed of 13 genes; namely, *CEACAM6, CYP4B1, EIF2S2, HID1, IFFO1, MYO18A, PDCD2, SGIP1, SWSAP1*, and 4 unknown genes (*A_24_P127621, A_24_P683553, A_24_P255786, A_24_P916979*). The unknown genes, *A_24_P127621, A_24_P683553, A_24_P255786*, and *A_24_P916979*, have specific gene sequences and distinct chromosomal locations (chromosome 7, chromosome 9, chromosome 10, and chromosome X, respectively); however, there was no known functional annotation of them in any of the annotation sources. Further in-depth experimental research studies might be performed to elucidate their function and association with PA tumorigenesis and/or invasiveness. The high prognostic power of the core module was not specific to NFPA but also observed in cancers with the same tissue origin such as glandular-origin cancers and brain cancers.

Among the module genes, *CEACAM6* encodes a protein that is a member of the carcinoembryonic antigen (CEA) family whose crew are cell surface glycoproteins (33). Members of this family have a function in cell adhesion and they are widely employed as serum tumor markers for determinations of carcinoma (53). Moreover, over-expression of *CEACAM6* modulates cancer progression through aberrant cell differentiation, anti-apoptosis, cell growth, and resistance to therapeutic agents (54). In addition, *CEACAM6* over-expression in varied malignancies promoting cell invasion and metastasis, therefore, it represents a characteristic advantage of tumor cells which are responsible for an invasive phenotype (55). It is also a major determinant for malignant phenotype of pancreatic cancer through over-expression of the positive regulation of epithelial–mesenchymal transition (35, 36, 56).

*CYP4B1* gene encodes a member of the cytochrome P450 superfamily of enzymes which is responsible for the oxidation of steroids, fatty acids, and xenobiotics therefore involved in reactions in drug metabolism (37). *CYP4B1* was reported as procarcinogen for some chemicals in bladder carcinoma, down-regulated in esophageal squamous cell carcinoma and up-regulated in breast tumors when comparing the surrounding healthy tissues (38, 57, 58).

*EIF2S2* encodes eukaryotic translation initiation factor 2 and functions as a vehicle in the early phases of protein synthesis. The deletion of *EIF2S2* gene has been reported with suppression of testicular germ cell tumor incidence and recessive lethality in mice (39). It is also reported that *EIF2S2* is up-regulated and involved in major enriched genes in epithelial ovarian carcinoma (EOC) samples and cervical cancer vs. normal group (53, 59, 60). In ER-negative breast cancer, *EIF2S2* gene was found as a member of novel loci (61).

*HID1* gene encodes a protein functioning in the trafficking of cargos that are important for the sorting/biogenesis/maturation of dense core vesicles and plays an important role in the development of cancers in a broad range of tissues (40, 62). It is alternatively named as *DMC1* (down-regulated in multiple cancer 1). The loss of expression of *HID1* was reported in breast, cervical, lung, thyroid, renal, and gastrointestinal cancer cell lines (62).

*IFFO1* gene is a member of the intermediate filament family which include essential components of the cytoskeleton and nuclear envelope (41, 42). In ovarian cancer, its promoter was hypermethylated and *IFFO1* was proposed as a biomarker (63). In addition, it has been identified as a strong prognostic indicator of breast cancer (63). The hypomethylation and up-regulation of *IFFO1* have been also reported in endometrioid endometrial adenocarcinoma samples (64).

*MYO18A* encodes a protein which is part of a complex that unifies lamellar actomyosin bundles and needed for cell migration. MYO18A has been found highly expressed in metastatic prostate cancer and its knockdown affects the cytoskeleton and cell migration (65). *MYO18A* gene fusion has been identified in an acute cell leukemia patient (66).

*PDCD2* (programmed cell death 2) gene encodes a nuclear protein expressed in a variety of tissues and its expression is controlled by transcriptional repressor *BCL6*. It plays an important role in cell death and/or in the regulation of cell proliferation. *PCDC2* has been found as a tumor suppressor and involved in the pathogenesis of osteosarcoma. Aberrant expression of *PDCD2* is associated with many tumors, such as leukemia and gastric cancer (44, 67). *PDCD2* is an important predictor of clinical relapse in acute leukemia patients (67). Plus, loss of *PDCD2* expression could induce gastric cancer development and progression through cell growth arrest at the early S phase of the cell cycle and reported as a putative tumor suppressor in gastric stromal tumors (68).

*SGIP1* (SH3 domain GRB2 like endophilin interacting protein) functions as an endocytic protein that has effects on signaling in neuronal systems including energy homeostasis (45, 46). Hypomethylation and retrotransposition of *SGIP1* have been reported in colorectal cancer samples (69). *SGIP1* has been found as a potential therapeutic target for obesity- and diabetes-related symptoms, since the selective reduction of the expression of SGIP1 consequenced with inhibition of food intake and the decrement of body weight in rat models (70). *SGIP1* has been identified as the second most significantly upregulated gene in the colon and rectal cancers samples (71). The expression level of *SGIP1* has been shown significantly decreased in gastric cancer when compared to control samples (72).

*SWSAP1* (SWS1-Associated Protein 1) gene is involved in homologous recombination repair by binding single-stranded DNA activity and it also has ATPase activity. Depletion of *SWSAP1* contributes to defects in homologous recombination repair and Knockdown of *SWSAP1* ends up with increased cell sensitivity to the DNA damaging (47).

Remaining core module gene members (*A_24_P127621, A_24_P255786, A_24_P683553, A_24_P916979*) were unknown with their unknown functions. The present study may contribute to assigning function/ functions to these genes. Known module genes were briefly explained with their functions and roles in various types of cancers. But there was no research found that could report module members for PA progression and prognosis. Therefore, identified module genes and module itself are novel for PA prognosis and they might be considered as the indicator of the non-invasiveness.

While our study provides some insight into the prognosis of PA in different states, more efforts should be performed to validate them clinically and extend our findings. We examined the prognosis of PA through core module genes in the context of gene co-expression networks. Results were *in silico* validated through various cancer types and it can be concluded that the core module is novel for PA prognosis. In addition, core module has also found as prognostic for brain tumors (glioblastoma, meningioma, and medulloblastoma) and glandular- originated tumors (adrenocortical carcinoma, breast invasive carcinoma, and ovarian serous cystadenocarcinoma). Our findings are based on TCGA, ArrayExpress, and GEO data (module investigation and validation), so a critical extension of this work would be to learn whether the patterns can be recapitulated by clinical trials. All in all, future efforts should be carried out to incorporate this systems-level understanding of prognostic genes into the practice of construction of effective clinical prognostic vehicles.

## Conclusion

Pituitary adenomas are the most common intracranial tumors in the central nervous system. They are accepted as benign in general but somehow tumors can exhibit gross invasion into surrounding tissues rarely. Invasion results in resistance to conventional treatment methods and leading to early and frequent recurrences. In order to shed light on the multi-layered molecular mechanism lay behind the invasion of PA, there is a great need for omics-level data and their integration into meta-analyses. Our study used gene co-expression analysis to construct a gene co-expression network, elucidate and validate network modules associated with the invasiveness and prognosis of PA. Eventually, a core module with 13 genes including *CEACAM6, CYP4B1, EIF2S2, HID1, IFFO1,MYO18A, PDCD2, SGIP1, SWSAP1*, and 4 unknown genes (*A_24_P127621, A_24_P255786, A_24_P683553, A_24_P916979*) were identified and *in silico* validated in association with the indicator of invasiveness and prognosis of PA, plus some related cancer types. The research not only increases the theoretical knowledge but also provides a novel prognostic module and therapeutic strategy for PA.

## Data Availability

Publicly available datasets were analyzed in this study. This data can be found here: https://www.ebi.ac.uk/arrayexpress/experiments/E-TABM-899/.

## Author Contributions

BA and KYA designed the data analysis framework. BA performed the data analyses and evaluated the results. KYA conceived and directed the study. BA drafted the manuscript. KYA revised the manuscript. All authors read and approved the final manuscript.

### Conflict of Interest Statement

The authors declare that the research was conducted in the absence of any commercial or financial relationships that could be construed as a potential conflict of interest.

## Supplementary Material

Figure S1 Prognostic performance of core module genes through lung and prostate cancers. Survival analyses indicated insignificant prognostic performance in both lung (*p* = 0.08) and prostate (*p* = 0.15) carcinomas.
