# Supplementary figures and images for: Co-expression Network Analysis Elucidated a Core Module in Association With Prognosis of Non-functioning Non-invasive Human Pituitary Adenoma
Source: Front Endocrinol (Lausanne). 2019 Jun 6;10:361. doi: 10.3389/fendo.2019.00361 (PMC6563679; doi:10.3389/fendo.2019.00361)

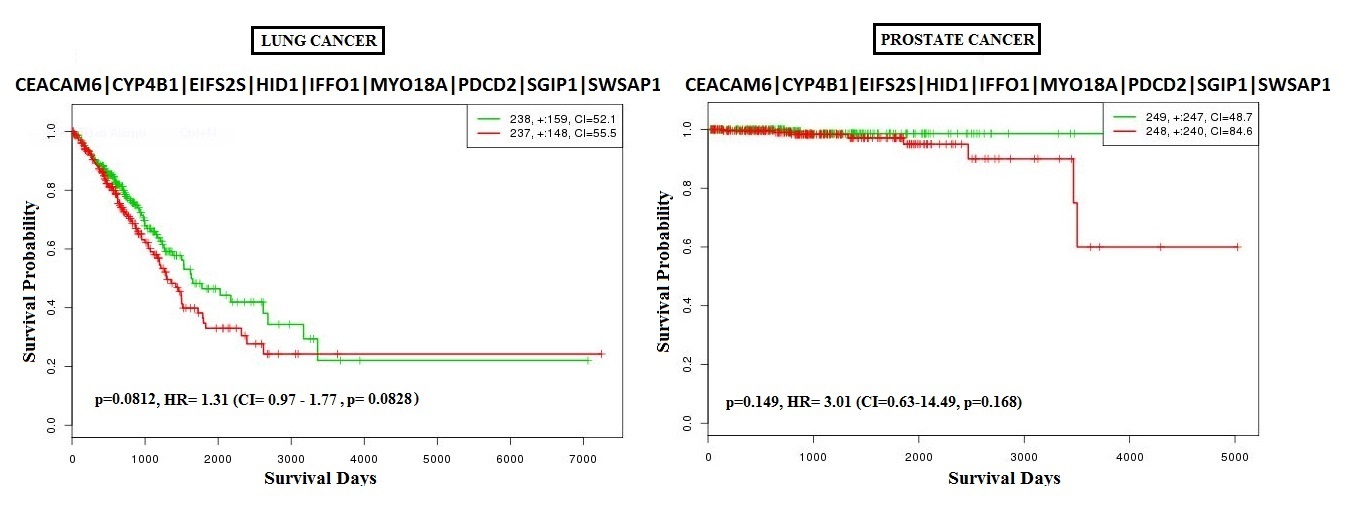

Supplement: Figure S1 — Prognostic performance of core module genes through lung and prostate cancers. Survival analyses indicated insignificant prognostic performance in both lung (p = 0.08) and prostate (p = 0.15) carcinomas. [file Image_1.JPEG]
